# Impact of iron status on kidney outcomes in kidney transplant recipients

**DOI:** 10.1038/s41598-023-28125-x

**Published:** 2023-01-17

**Authors:** Hyo Jeong Kim, Ro Han, Kyung Pyo Kang, Jung-Hwa Ryu, Myung-Gyu Kim, Kyu Ha Huh, Jae Berm Park, Chan-Duck Kim, Seungyeup Han, Hyung Woo Kim, Beom Seok Kim, Jaeseok Yang

**Affiliations:** 1 Department of Internal Medicine, Yonsei University College of Medicine, Seoul, Republic of Korea; 2 Department of Internal Medicine, Gachon University College of Medicine, Incheon, Republic of Korea; 3 Department of Internal Medicine, Research Institute of Clinical Medicine, Jeonbuk National University Medical School, Jeonju, Republic of Korea; 4 Biomedical Research Institute, Jeonbuk National University Hospital, Jeonju, Republic of Korea; 5 Department of Internal Medicine, Ewha Womans University Seoul Hospital, Seoul, Republic of Korea; 6 Department of Internal Medicine, Korea University College of Medicine, Seoul, Republic of Korea; 7 Department of Surgery, Yonsei University College of Medicine, Seoul, Republic of Korea; 8 Department of Surgery, Seoul Samsung Medical Center, Sungkyunkwan University, Seoul, Republic of Korea; 9 Department of Internal Medicine, Kyungpook National University Hospital, Daegu, Republic of Korea; 10 Department of Internal Medicine, Dongsan Medical Center, Keimyung University, Daegu, Republic of Korea; 11 Division of Nephrology, Department of Internal Medicine, College of Medicine, Yonsei University, 50 Yonsei-Ro, Seodaemun-Gu, Seoul, 03722 Republic of Korea

**Keywords:** End-stage renal disease, Risk factors, Transplantation

## Abstract

Iron plays an important role in hemodynamics and the immunity, independent of anemia. Since dynamic changes occur in iron storage after kidney transplantation (KT), we investigated the association between iron status and kidney outcomes in KT patients. We analyzed data from the KoreaN cohort study for Outcome in patients With KT (KNOW-KT). The iron status was classified into three groups based on ferritin or transferrin saturation (TSAT) levels one year after KT, with reference ranges of 20‒35% and 100‒300 ng/mL for TSAT and ferritin, respectively. The primary outcome was the composite outcome, which consisted of death, graft failure, and an estimated glomerular filtration rate decline ≥ 50%. In total, 895 patients were included in the final analysis. During a median follow-up of 5.8 years, the primary outcome occurred in 94 patients (19.8/1000 person-years). TSAT levels decreased one year after KT and thereafter gradually increased, whereas ferritin levels were maintained at decreased levels. The adjusted hazard ratios (95% confidence intervals) for the composite outcome were 1.67 (1.00–2.77) and 1.20 (0.60–2.40) in the TSAT > 35% and ferritin > 300 ng/mL groups, respectively. High iron status with high TSAT levels increases the risk of graft failure or kidney functional deterioration after KT.

## Introduction

The prevalence of end-stage kidney disease (ESKD) is increasing worldwide^1^. Kidney transplantation (KT) is the preferred treatment over dialysis for ESKD since KT shows lower mortality and morbidity with better quality of life than dialysis^2^. According to the United States Renal Data System annual data report, the cumulative number of KT recipients reached 22,393, which is an increase of 6.5% since 2017^1^. The development of surgical techniques and immunosuppressive drugs has markedly improved short-term graft survival in KT recipients^3^. However, there has been little improvement in the long-term outcomes^4,5^. Therefore, further efforts other than optimizing immunosuppression are needed to improve KT outcomes.

Anemia is a common complication in patients with chronic kidney disease (CKD) and also has a high prevalence in KT recipients^6^. According to recent studies, post-KT anemia shows a biphasic pattern. The prevalence of anemia was found to be 76% at the time of KT and 21% and 36% after one and four years of transplantation, respectively^7^. Iron deficiency is the most important cause of anemia during the first year after KT, while impaired renal function plays an important role in causing anemia thereafter^6,8^. Iron storage undergoes dynamic changes after KT. Transferrin saturation (TSAT) and ferritin levels, the parameters reflecting iron status, showed a decreasing pattern during the recovery from anemia after KT^9^. Several factors such as surgical blood loss, frequent blood sampling, inadequate nutrition, and increased erythropoietin production by kidney graft can decrease iron storage^8,10^. Medications such as immunosuppressants also influence iron metabolism by increasing hepcidin expression and ferroportin degradation, reducing iron absorption^11^. Moreover, infection and systemic inflammation are also known to affect iron metabolism. Interestingly, almost 10% of KT recipients continued to have iron overload status even after 3 years of KT, although overall iron storage showed decreasing patterns after KT^12^.

An increasing body of evidence suggests an association between iron dysregulation and adverse kidney outcomes. Recent studies have shown an association between iron deficiency and ESKD risk in patients with CKD regardless of anemia^13,14^. Moreover, iron induces oxidative stress and inflammation in tissues, increasing the risk of CKD progression^13,15^. Iron accumulation activates the renal renin-angiotensin system, increasing inflammation and fibrosis, ultimately accelerating diabetic nephropathy^16^. Although iron homeostasis also plays an essential role in kidney outcomes in those who have not undergone KT, the association between iron status and graft outcomes in KT recipients is controversial^17–20^. Herein, we investigated the iron status based on TSAT and ferritin levels one year after KT and its association with kidney outcomes using a Korean multicenter KT cohort.

## Results

### Baseline characteristics of study population according to iron status

The clinical characteristics of the 895 patients stratified by TSAT level one year after KT are presented in Table 1. TSAT levels classified 253 (28.2%), 385 (43.0%), and 257 (28.7%) patients into groups with TSAT levels ≤ 20%, 21–35%, and > 35%, respectively. Females and diabetic patients tended to have lower TSAT levels. Additionally, the low TSAT group had lower hemoglobin and ferritin levels one year after KT.

**Table 1:** Baseline characteristics of patients with respect to the TSAT.

	Total (N = 895)	TSAT			*P*
		≤ 20% (N = 253)	21–35% (N = 385)	> 35% (N = 257)	
Demographic data					
Recipient age, years	46.1 ± 11.4	45.6 ± 11.3	46.2 ± 11.6	46.4 ± 11.3	0.710
Recipient female, n (%)	326 (36.4)	115 (45.5)	132 (34.3)	79 (30.7)	**0.001**
Donor age, years	45.6 ± 12.0	45.2 ± 12.4	45.4 ± 11.9	46.2 ± 11.8	0.620
Donor female, n (%)	434 (48.5)	120 (47.4)	187 (48.6)	127 (49.4)	0.900
Systolic blood pressure, mmHg	137.8 ± 19.4	135.7 ± 18.7	138.3 ± 19.9	139.0 ± 19.3	0.100
Body mass index, kg/m^2^	22.9 ± 3.4	23.0 ± 3.7	23.1 ± 3.4	22.5 ± 3.2	0.110
Smoking history, n (%)					0.320
Never	465 (52.0)	137 (54.2)	198 (51.4)	130 (50.6)	
Current	67 (7.5)	12 (4.7)	30 (7.8)	25 (9.7)	
Former	363 (40.6)	104 (41.1)	157 (40.8)	102 (39.7)	
Alcohol history, n (%)					0.440
Never	184 (20.6)	57 (22.5)	69 (17.9)	58 (22.6)	
Moderate drinker	281 (31.4)	81 (32.0)	119 (30.9)	81 (31.5)	
Heavy drinker	430 (48.0)	115 (45.5)	197 (51.2)	118 (45.9)	
Primary renal disease, n (%)					0.340
Diabetic nephropathy	192 (21.5)	60 (23.7)	89 (23.1)	43 (16.7)	
Hypertensive nephropathy	211 (23.6)	58 (22.9)	86 (22.3)	67 (26.1)	
Glomerulonephritis	267 (29.8)	80 (31.6)	111 (28.8)	76 (29.6)	
Polycystic kidney disease	47 (5.3)	12 (4.7)	21 (5.5)	14 (5.4)	
Others	178 (19.9)	43 (17.0)	78 (20.3)	57 (22.2)	
Transplantation information					
ABO incompatible, n (%)	157 (17.5)	43 (17.0)	66 (17.1)	48 (18.7)	0.850
HLA mismatch count n (%)					
0	37 (4.1)	11 (4.3)	14 (3.6)	12 (4.7)	0.930
1–3	434 (48.5)	119 (47.0)	192 (49.9)	123 (47.9)	
4–6	424 (47.4)	123 (48.6)	179 (46.5)	122 (47.5)	
Immunosuppressant, n (%)					**0.015**
Tacrolimus	837 (93.5)	236 (93.3)	352 (91.4)	249 (96.9)	
Cyclosporine	39 (4.4)	8 (3.2)	25 (6.5)	6 (2.3)	
Others	19 (2.1)	9 (3.6)	8 (2.1)	2 (0.8)	
Living donor, n (%)	732 (81.8)	212 (83.8)	322 (83.6)	198 (77.0)	0.065
Comorbidities					
Hypertension, n (%)	835 (97.8)	229 (97.0)	366 (98.7)	240 (97.2)	0.310
Diabetes, n (%)	309 (36.2)	98 (41.5)	132 (35.6)	79 (32.0)	0.088
Hepatitis B virus, n (%)	194 (21.9)	58 (23.6)	80 (20.9)	56 (21.8)	0.740
Hepatitis C virus, n (%)	10 (1.2)	2 (0.8)	5 (1.4)	3 (1.2)	0.830
Laboratory parameters					
Hemoglobin at transplantation, g/dl	10.6 ± 1.6	10.5 ± 1.5	10.6 ± 1.7	10.6 ± 1.6	0.610
Hemoglobin after 1 year, g/dl	13.6 ± 1.9	13.1 ± 2.0	13.7 ± 1.9	13.9 ± 1.7	** < 0.001**
Albumin after 1 year, g/dl	4.4 ± 0.3	4.4 ± 0.3	4.4 ± 0.3	4.4 ± 0.4	0.610
Total cholesterol after 1 year, mg/dl	178.3 ± 35.6	178.9 ± 37.3	179.6 ± 35.5	175.6 ± 33.8	0.350
eGFR after 1 year, ml/min/1.73 m^2^	64.5 ± 18.1	66.5 ± 20.4	63.4 ± 17.8	64.1 ± 16.1	0.100
Ferritin at transplantation, ng/dl	197.2 ± 222.0	149.6 ± 153.6	193.4 ± 171.4	250.0 ± 316.1	** < 0.001**
Ferritin after 1 year, ng/dl	138.6 ± 193.2	85.5 ± 137.1	135.3 ± 140.7	195.7 ± 275.8	** < 0.001**
TSAT at transplantation, %	34.0 ± 18.8	31.1 ± 17.9	33.0 ± 17.5	38.3 ± 20.6	** < 0.001**
TSAT after 1 year, %	28.8 ± 14.3	13.2 ± 4.6	27.4 ± 4.4	46.3 ± 10.6	** < 0.001**
CRP after 1 year, mg/dl	0.18 (0.03–0.50)	0.20 (0.04–0.60)	0.15 (0.03–0.50)	0.28 (0.03–0.50)	0.150

Data are presented as the mean ± standard deviation, number (percentage), or median (interquartile range). Significant values are in bold.

*SD* standard deviation, *HLA* Human leukocyte antigen, *eGFR* estimated glomerular filtration rate, *TSAT* transferrin saturation, *CRP* c-reactive protein.

The baseline characteristics according to ferritin levels at one year after KT are shown in Supplementary Table S1. According to the ferritin levels at one year after KT, 512 (57.2%), 302 (33.7%), 81 (9.1%) patients were classified into the low ferritin group (≤ 100 ng/ml), an intermediate ferritin group (101–300 ng/ml), and a high ferritin group (> 300 ng/ml), respectively. Participants in the low ferritin group, had more living donors and were characterized by higher hemoglobin, lower TSAT, and lower CRP levels one year after KT.

### Temporal changes in iron status and anemia after kidney transplantation

**Figure 1:**
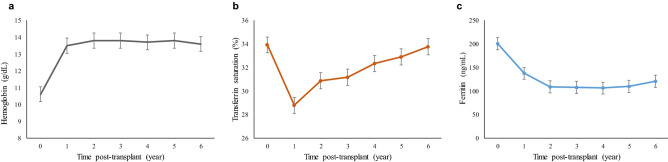
Temporal changes of hemoglobin and iron status. (**A**) Hemoglobin, (**B**) transferrin saturation, (**C**) Ferritin.

**Figure 2:**
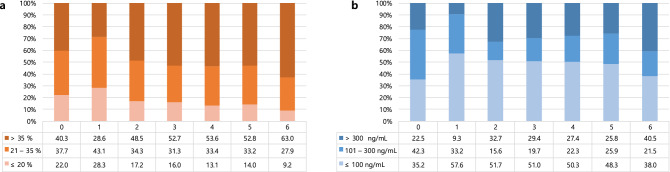
Temporal changes of iron groups according to transferrin saturation levels (**A**) and ferritin levels (**B**).

The iron status from KT to six years after KT is shown in Fig. 1. The anemia improved and hemoglobin levels reached a plateau one year after KT, increasing from 10.6 ± 1.6 g/dl to 13.6 ± 1.9 g/dl (Fig. 1a). During the first year post KT, TSAT levels showed a decreasing trend and gradually returned to the pre-transplant value (Fig. 1b). Ferritin levels decreased gradually after KT, and the value was almost halved after six years (Fig. 1c). When iron groups according to TSAT levels were followed up from pre-KT time to six years after KT, proportions of the low TSAT group increased one year after KT and then decreased two years after KT, while those of the high TSAT group showed an opposite trend (Fig. 2a). Proportions of the high ferritin group had decreased one year after KT and subsequently increased two years after KT (Fig. 2b).

### Outcome event rates according to iron status

The outcome event rates according to the iron statuses are presented in Table 2. During 4740.8 person-years of follow-up (median 5.8 years), a total 94 (10.5%, 19.8 per 1,000 person-years) composite outcome events occurred. The number of event occurrences in TSAT groups ≤ 20%, 21–35%, and > 35% were 33 (3.7%), 31 (3.5%), and 30 (3.4%), respectively. The corresponding incidence rates were 24.6, 15.2, and 22.1 per 1000 person-years, respectively. In addition, the number of event occurrences in the ferritin groups ≤ 100 ng/ml, 101–300 ng/ml, and > 300 ng/ml were 47 (5.3%), 36 (4.0%), and 11 (1.2%), respectively.

**Table 2:** Outcome event rates with respect to the TSAT, ferritin, and iron status classification.

	Number of person-years	Number of events (%)	Incidence rate^a^
Total (N = 895)	4740.8	94 (10.5)	19.8
TSAT			
≤ 20%	1343.7	33 (3.7)	24.6
21–35%	2041.7	31 (3.5)	15.2
> 35%	1355.4	30 (3.4)	22.1
Ferritin			
≤ 100 ng/ml	2659.9	47 (5.3)	17.7
101–300 ng/ml	1654.6	36 (4.0)	21.8
> 300 ng/ml	426.3	11 (1.2)	25.8

^a^Per 1000 person-years.

The primary outcome was composite of all-cause mortality, graft failure, and eGFR ≥ 50%. Graft failure is defined as the development of kidney failure requiring dialysis or KT, or death with a functional graft.

*TSAT* transferrin saturation, *eGFR* estimated glomerular filtration rate, *KT* kidney transplantation.

### Association between iron parameters and primary outcomes

Recipient and donor age, recipient and donor sex, type of transplantation (living or deceased donor), history of diabetes mellitus, smoking history, and laboratory tests including hemoglobin, albumin, and eGFR one year after KT were used as covariates in the multivariate Cox regression analysis. The high TSAT group was associated with a higher risk of composite outcomes (Table 3). In patients in the TSAT > 35% group, the risk of composite outcomes significantly increased compared that for the TSAT 20–35% group (adjusted HR 1.67; 95% CI 1.00–2.77). However, analysis with different classifications by using ferritin levels showed no significant differences (adjusted HR 1.20; 95% CI 0.60–2.40).

**Table 3:** Adjusted hazard ratios for outcomes based on iron parameters in multivariate Cox regression analysis (Multivariate analysis was adjusted for recipient and donor age, recipient and donor’s sex, type of transplantation (living donor or deceased donor), history of diabetes mellitus, smoking history, and laboratory tests including hemoglobin, albumin, and estimated glomerular filtration rate at 1 year after kidney transplantation).

	HR	95% CI	P-value
TSAT			
≤ 20%	1.40	0.84–2.33	0.193
21–35%	*Reference*		
> 35%	1.67	1.00–2.77	**0.049**
Ferritin			
≤ 100 ng/mL	0.85	0.53–1.37	0.500
101–300 ng/mL	*Reference*		
> 300 ng/mL	1.20	0.60–2.40	0.608

The primary outcome was composite of all-cause mortality, graft failure, and eGFR ≥ 50%. Graft failure is defined as the development of kidney failure requiring dialysis or KT, or death with a functional graft. Significant values are in bold.

*HR* hazard ratio, *CI* confidence interval, *TSAT* transferrin saturation, *eGFR* estimated glomerular filtration rate, *KT* kidney transplantation.

### Association between iron parameters and secondary outcomes

In the secondary outcome analyses, there was no statistically significant association with iron parameters (Supplementary Table S2). Although there was no statistically significant association between iron parameters and a ≥ 50% eGFR decline, there was a similar trend as the composite outcome; adjusted HR (95% CI) 1.39 (0.80–2.39), 1.53 (0.88–2.67) in TSAT ≤ 20%, > 35% groups, respectively.

## Discussion

In this study, we investigated the association between iron status and the risk of composite adverse kidney outcomes. We found that both TSAT and ferritin levels initially decreased with improving anemia after KT, and then TSAT increased again. Further, the TSAT ˃ 35% group had a higher risk of adverse kidney outcomes independent of anemia, compared to the TSAT 21–35% group. Further analyses of secondary outcomes showed no statistical significant results on rejection, mortality, graft failure, and ≥ 50% eGFR decline. Our findings suggest that KT recipients with high iron status may be more vulnerable to adverse kidney outcomes or kidney function decline.

Iron is an essential micronutrient in energy metabolism, cell death, and immune regulation^21–23^. Iron plays a crucial role in oxygen transport in the body, and the delivered oxygen is involved in various energy metabolic processes^24^. In the case of iron overload, excess iron exists in the form of a non-transferrin bound form, participating in the redox cycles^25–27^. Oxidative stress through the Fenton reaction impairs proteins, deoxyribonucleic acid, and cell membranes. High iron also increases the risk of atherosclerotic plaque formation, infection, and malignant cell growth, eventually leading to a higher mortality risk^28–30^. Through these mechanisms, high iron might have contributed to a higher mortality in KT patients.

Iron is involved in both innate and adaptive immune systems^31,32^. Macrophages play a vital role in iron regulation, and 95% of the iron used for metabolism depends on the macrophage-iron recycling system^33,34^. Reciprocally, iron promotes macrophage polarization to secrete pro-inflammatory cytokines such as TNF-α, IL-1β, and macrophages ultimately acquire bacterial resistance^35–38^. In addition, iron regulates T-cell proliferation, differentiation, and maturation^37,39^. Since regulation of the immune system is critical for kidney allograft survival, it can be inferred that superfluous iron may promote immune response, contributing to allograft rejection and failure. In a rodent-model experiment, iron overload altered the composition of immune subsets and shortened graft survival in heart transplanted rats^40^.

Kidney disease progression is accelerated by iron overload through the induction of inflammation and fibrosis via oxidative stress and activation of the renin-angiotensin system^13,15,16^. In an experimental study, administration of an iron-chelating agent attenuated interstitial fibrosis in CKD rats^41^. Furthermore, rats with an iron-restricted diet showed reduced inflammatory cytokines and extracellular matrix mRNA expression, protecting the kidney^42^. These studies suggest the clinical significance of iron overload in renal functional deterioration in CKD patients including KT patients.

Few studies have been conducted about role of iron overload in KT patients and their results are controversial. A previous study showed that iron, TSAT, and ferritin were not associated with graft survival and mortality^20^. In other studies, high ferritin levels before KT had a favorable prognosis for kidney allografts by protecting against renal ischemia–reperfusion injury^43^. The discrepancy among studies was probably caused by different definitions of iron status and different measurement times of iron parameters among studies with small sample sizes.

Our study investigated the association between iron status and graft outcomes using iron parameters measured one year after KT. We tried to assess iron status at a stable period after KT instead of assessing during the KT operation or immediately before KT. Kidney allograft functions gradually stabilize six months to one year after KT, and we used one year after KT as a baseline to minimize the possibility of abnormal iron status due to decreased allograft function. With this background, our study showed that TSAT ˃ 35% was associated with the increased risk of adverse kidney graft outcomes, irrespective of anemia. When association of TSAT with rejection or separate outcomes of the composite outcome, there was no significant association for rejection, graft failure, or mortality. However, there was still a similar trend of association between TSAT and ≥ 50% eGFR decline despite not reaching statistical significance, suggesting that iron status may mainly affect renal functional decline rather than rejection or mortality.

Ferritin is generally considered a more sensitive marker of iron deficiency than TSAT, since ferritin levels decrease before TSAT. Therefore, ferritin is emphasized in the evaluation of iron stores and is an independent predictor of clinical outcomes. On the other hand, several previous studies suggest that TSAT level is a good indicator of iron overload, supporting its role as a prognostic factor^44,45^. Our study also showed a positive association between TSAT levels and adverse kidney outcomes, possibly because TSAT rises earlier than ferritin in an iron-sufficient environment^46^, indicating that TSAT could be a more sensitive indicator for evaluating the prognosis of transplanted kidney function in an iron overload status. Furthermore, iron parameters can be affected by other factors such as inflammation and malnutrition, resulting in increased serum ferritin and decreased TSAT levels. Therefore, it is challenging to explain iron overload with an increase in ferritin levels since inflammation might increase ferritin levels even in an iron-deficient environment^47^.

Although advances in erythropoietin therapy are expected to reduce iron overload in patients with CKD, iron overload is still prevalent, and is approximately 36% in the case of ESKD^48,49^. Moreover, the prevalence and cause of iron overload after KT are not fully understood. We found that the prevalence of iron overload initially decreased after KT. This could be attributed to endogenous erythropoietin production by functioning kidney allograft, which mobilizes iron stores for hemoglobin synthesis^12^. Subsequently, the prevalence of iron overload increased after 2 years following KT. It is proposed that iron supplementation, blood transfusion before KT, and the genetic mutations *HFE* C282Y and *HFE* H63D contributed to iron overload^12^. Consistent with this, recent studies demonstrated that patients with iron excess had more iron supplements before KT^50^. Since excessive iron accumulates in multiple organs with no metabolic process to remove this accumulated iron, precautions are needed when using intravenous iron and erythropoietin therapies. Therefore, our study supports the importance of iron status follow-up and recommends avoiding unnecessary supplements before and after KT.

Based on the study results that TSAT and ferritin levels rapidly declined until one year after KT and then increased and formed a plateau approximately 2 years after KT, we recommend annual iron status check-up until 2 years after KT. After then, interval of ferritin and TSAT measurement should be individualized according to the condition of KT patients, such as infection, the amount and frequency of blood transfusion, graft functions, and nutritional status. Once iron overload is diagnosed, we suggest starting treatment to prevent adverse kidney outcomes. We can consider iron chelating agents for transfusion-associated iron overload with cautious monitoring of kidney function^51^. In cases of genetic hemochromatosis or iron overload without anemia caused by immunosuppressant drug use, obesity, or prior use of iron replacement, phlebotomy or erythropoietin can be a treatment option^52,53^.

Our study had several limitations. Firstly, this observational cohort study lacked detailed information on transfusions, history of bleeding, proteinuria measurements, and medications including iron supplements and erythropoietin injections. Consequently, it was impossible to precisely analyze the causes of iron overload after KT. Secondly, potential confounding factors cannot be completely controlled in this observational study. Although adjustments for albumin and C-reactive protein were tried to screen out highly inflammatory status as a cause of high ferritin, we cannot completely exclude causes of high ferritin other than iron overload due to lack of thorough clinical information about these factors. Lastly, the follow-up duration of this study was not long enough to analyze the long-term impact of iron status after KT. Further studies, including this cohort study at later points, are required to elucidate this long-term impact. Despite its limitations, this study demonstrated temporal changes in iron status after KT. Furthermore, the study explains the association between iron status and kidney allograft function, and therefore has significance as a cornerstone for more controlled clinical trials on a larger scale.

In conclusion, high iron status, represented by a high TSAT level, is significantly associated with an increased risk of adverse outcomes in KT recipients, independent of anemia.

## Methods

### Study participants

The KoreaN cohort study for Outcome in patients With Kidney Transplantation (KNOW-KT) is a prospective, observational, cohort study conducted by eight transplant centers in South Korea^54^. Patients were enrolled in KNOW-KT from July 2012 to August 2016 and have been followed up annually until now. The protocol summary is registered in the International Clinical Trial Registry (NCT02042963). All study procedures were performed in accordance with the Declaration of Helsinki guidelines and the study was approved by the institutional review boards of the participating clinical centers (IRB 2021-4265-001). Informed consent was obtained from all study participants.

**Figure 3:**
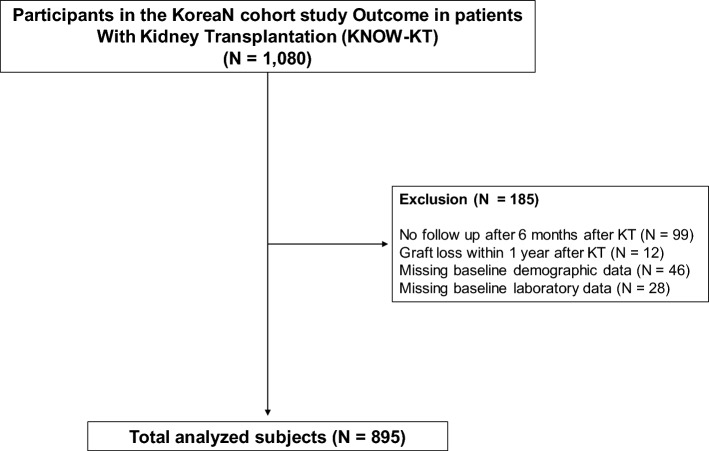
Flow diagram of the study cohort. *KT* kidney transplantation.

In total, 1,080 KT recipients were enrolled. We excluded 99 patients who had no follow-up six months after KT and 12 who underwent graft loss within one year after KT. Moreover, 46 patients with missing baseline demographic data and 28 patients without baseline laboratory data were excluded. Finally, 895 patients were included in the study (Fig. 3).

### Data collection and measurements

The baseline demographics, anthropometric measurements, and comorbid status of both recipients and donors were collected during the pre-transplant screening. Baseline laboratory tests, including hemoglobin, creatinine, albumin, iron profiles (serum iron, TSAT, ferritin), total cholesterol, and C-reactive protein (CRP), were measured at each visit. Serum creatinine level was measured using an isotope-dilution mass spectrometry-traceable method^55^, and the eGFR was calculated using the four-variable Modification of Diet in Renal Disease formula^56^.

### Main predictors

The main predictors of this study were iron status, defined using TSAT, and ferritin levels measured one year after KT. Since the iron status as well as renal functions stabilize one year after KT, we selected it as a baseline in our study. TSAT was calculated as the percentage of the ratio of serum iron levels divided by the total iron binding capacity. Ferritin and TSAT levels were classified into three groups, where 20%, and 35% were the reference points for the TSAT, and 100 ng/ml and 300 ng/ml were the reference points for ferritin.

### Study outcomes

The primary outcome was a composite outcome of all-cause mortality, graft loss, and a ≥ 50% decline in eGFR. Degree of eGFR decline was calculated as eGFR decline from the eGFR value at 1 year after KT as the baseline. Graft loss was defined as the development of kidney failure that required dialysis or KT. The secondary outcome included separate outcomes from the primary outcome, which were all-cause mortality, graft loss, and a ≥ 50% decline in eGFR. Rejection was also included in our secondary outcome, which was defined as biopsy-confirmed acute or chronic rejection. Survival time was defined as the time from one year after transplantation to the date of composite event occurrence. Patients who failed to follow-up were censored at the time of their last visit.

### Statistical analyses

Continuous variables are presented as mean ± standard deviation, while categorical variables are presented as numbers and proportions. Categorical variables were compared using the chi-squared test or Fisher’s exact test, as appropriate. The Kolmogorov–Smirnov test was used to confirm the normality of the distribution. Continuous variables with a normal distribution were compared using the analysis of variance test. Variables with skewed distributions are presented as medians with interquartile ranges (IQR) and compared using the Kruskal–Wallis test. Furthermore, Cox proportional hazard regression models were used to investigate the association between iron status and composite outcomes. Univariate models were first used in the primary analysis. Subsequently, multivariate models were used with covariates having a significance level of < 0.1 in the univariate analysis. The results were presented as hazard ratios (HRs) and 95% confidence intervals (CIs). Statistical significance was defined as *P* < 0.05. All statistical analyses were performed using Stata version 15 (StataCorp LLC, College Station, TX, USA).

## Supplementary Information

Supplementary Tables.

## Data Availability

The data are not publicly available since the ownership belongs to Seoul National University Hospital Medical Research Cooperation Center. However, the data will be shared on reasonable request to the corresponding author.
