# Supplementary material for: Impact of iron status on kidney outcomes in kidney transplant recipients
Source: Sci Rep. 2023 Jan 17;13:861. doi: 10.1038/s41598-023-28125-x (PMC9845230; doi:10.1038/s41598-023-28125-x)
Supplement: Supplementary file 1 — Supplementary Tables. [file 41598_2023_28125_MOESM1_ESM.pdf]

SUPPLEMENTARY MATERIAL

Impact of Iron Status on Kidney Outcomes in Kidney Transplantation Patients

Corresponding author: Jaeseok Yang (jcyjs@yuhs.ac)

TABLE OF CONTENTS

Supplementary Table S1. Baseline characteristics of patients with respect to the ferritin

Supplementary Table S2. Adjusted hazard ratios for secondary outcomes based on iron parameters in multivariate Cox regression analysis

Table S1. Baseline characteristics of patients with respect to the ferritin

	Total<br>N = 895	Ferritin<br>≤ 100 ng/mL<br>N = 512	Ferritin<br>101 – 300 ng/mL<br>N = 302	Ferritin<br>> 300 ng/mL<br>N = 81	*P*
**Demographic data**					
Recipient age, years	46.1 ± 11.4	46.0 ± 11.6	45.8 ± 11.3	48.0 ± 10.1	0.290
Recipient female, n (%)	326 (36.4)	180 (35.2)	109 (36.1)	37 (45.7)	0.190
Donor age, years	45.6 ± (12.0	44.9 ± 12.0	46.2 ± 11.8	47.7 ± 12.7	0.086
Donor female, n (%)	434 (48.5)	253 (49.4)	145 (48.0)	36 (44.4)	0.690
Systolic blood pressure, mmHg	137.8 ± 19.4	135.9 ± 18.4	140.1 ± 20.0	141.2 ± 21.9	**0.003**
Body mass index, kg/m^2	22.9 ± 3.4	22.9 ± 3.5	23.0 ± 3.3	22.1 ± 3.3	0.099
**Smoking history, n (%)**					0.094
Never	465 (52.0)	256 (50.0)	164 (54.3)	45 (55.6)	
Current	67 (7.5)	46 (9.0)	13 (4.3)	8 (9.9)	
Former	363 (40.6)	210 (41.0)	125 (41.4)	28 (34.6)	
**Alcohol history, n (%)**					**0.037**
Never	184 (20.6)	120 (23.4)	49 (16.2)	15 (18.5)	
Moderate drinker	281 (31.4)	155 (30.3)	93 (30.8)	33 (40.7)	
Heavy drinker	430 (48.0)	237 (46.3)	160 (53.0)	33 (40.7)	
**Primary renal disease, n (%)**					**<0.001**
Diabetic nephropathy	192 (21.5)	115 (22.5)	57 (18.9)	20 (24.7)	
Hypertensive nephropathy	211 (23.6)	131 (25.6)	63 (20.9)	17 (21.0)	
Glomerulonephritis	267 (29.8)	176 (34.4)	76 (25.2)	15 (18.5)	
Polycystic kidney disease	47 (5.3)	27 (5.3)	17 (5.6)	3 (3.7)	
Others	178 (19.9)	63 (12.3)	89 (29.5)	26 (32.1)	
**Transplantation information**					
ABO incompatible, n (%)	157 (17.5)	78 (15.2)	62 (20.5)	17 (21.0)	0.110
HLA mismatch count n (%)					0.068
0	37 (4.1)	22 (4.3)	11 (3.6)	4 (4.9)	
1 – 3	434 (48.5)	259 (50.6)	148 (49.0)	27 (33.3)	
4 – 6	424 (47.4)	231 (45.1)	143 (47.4)	50 (61.7)	
Immunosuppressant, n (%)					0.350
Tacrolimus	837 (93.5)	485 (94.7)	276 (91.4)	76 (93.8)	
Cyclosporine	39 (4.4)	19 (3.7)	16 (5.3)	4 (4.9)	

Others	19 (2.1)	8 (1.6)	10 (3.3)	1 (1.2)	
Living donor, n (%)	732 (81.8)	439 (85.7)	237 (78.5)	56 (69.1)	**<0.001**
**Comorbidities**					
Hypertension, n (%)	835 (97.8)	480 (97.8)	280 (97.2)	75 (100.0)	0.350
Diabetes, n (%)	309 (36.2)	181 (36.9)	99 (34.4)	29 (38.7)	0.700
Hepatitis B virus, n (%)	194 (21.9)	126 (25.0)	46 (15.3)	22 (27.5)	**0.002**
Hepatitis C virus, n (%)	10 (1.2)	5 (1.0)	4 (1.4)	1 (1.3)	0.900
**Laboratory parameters**					
Hemoglobin at transplantation, g/dL	10.6 ± 1.6	10.6 ± 1.6	10.5 ± 1.7	10.5 ± 1.5	0.430
Hemoglobin after 1 year, g/dL	13.6 ± 1.9	13.7 ± 1.9	13.6 ± 1.8	13.0 ± 2.1	**0.015**
Albumin after 1 year, g/dL	4.4 ± 0.3	4.4 ± 0.3	4.4 ± 0.3	4.5 ± 0.34	**0.014**
Total cholesterol after 1 year, mg/dL	178.3 ± 35.6	176.9 ± 34.7	179.0 ± 35.9	184.3 ± 39.0	0.200
eGFR after 1 year, mL/min/1.73 m^2	64.5 ± 18.1	65.9 ± 17.8	62.6 ± 17.9	62.7 ± 20.1	**0.026**
Ferritin at transplantation, ng/dL	197.2 ± 222.0	156.0 ± 140.2	211.0 ± 202.8	402.7 ± 460.1	**<0.001**
Ferritin after 1 year, ng/dL	138.6 ± 193.2	37.9 ± 25.2	204.7 ± 61.7	528.3 ± 404.4	**<0.001**
TSAT at transplantation, %	34.0 ± 18.8	33.3 ± 18.5	33.8 ± 17.5	39.6 ± 23.7	**0.020**
TSAT after 1 year, %	28.8 ±14.3	26.0 ± 14.0	32.2 ± 13.2	33.5 ± 15.78	**<0.001**
CRP after 1 year, mg/dL	0.18 (0.03 – 0.50)	0.09 (0.03 – 0.31)	0.40 (0.15 – 0.90)	0.30 (0.05 – 0.90)	**<0.001**

Note: Data are presented as the mean ± standard deviation, number (percentage), or median (interquartile range). Significant values are in bold.
Abbreviations: SD, standard deviation; HLA, Human leukocyte antigen; eGFR, estimated glomerular filtration rate; TSAT; transferrin saturation, CRP, c-reactive protein.

Table S2. Adjusted hazard ratios for secondary outcomes based on iron parameters in multivariate Cox regression analysis [a]

		HR	95% CI	P-value
**Mortality**				
	≤ 20 %	0.43	0.15 - 1.26	0.125
**TSAT**	21 – 35 %		*reference*	
	> 35 %	0.67	0.26 - 1.78	0.424
	≤ 100 ng/mL	0.65	0.25 - 1.71	0.380
**Ferritin**	101 – 300 ng/mL		*reference*	
	> 300 ng/mL	2.25	0.77 - 6.57	0.137
**Graft failure**				
	≤ 20 %	0.87	0.42 - 1.80	0.714
**TSAT**	21 – 35 %		*reference*	
	> 35 %	1.29	0.66 - 2.51	0.456
	≤ 100 ng/mL	0.85	0.44 - 1.61	0.610
**Ferritin**	101 – 300 ng/mL		*reference*	
	> 300 ng/mL	1.22	0.48 - 3.11	0.671
**eGFR ≥50% decline**				
	≤ 20 %	1.39	0.80 - 2.39	0.242
**TSAT**	21 – 35 %		*reference*	
	> 35 %	1.53	0.88 - 2.67	0.135
	≤ 100 ng/mL	1.07	0.63 - 1.80	0.808
**Ferritin**	101 – 300 ng/mL		*reference*	
	> 300 ng/mL	1.14	0.51 - 2.57	0.749
**Rejection**				
	≤ 20 %	0.80	0.49 - 1.29	0.355
**TSAT**	21 – 35 %		*reference*	
	> 35 %	1.33	0.88 - 2.01	0.173
	≤ 100 ng/mL	0.66	0.44 - 1.00	0.050
**Ferritin**	101 – 300 ng/mL		*reference*	
	> 300 ng/mL	1.02	0.55 - 1.90	0.954

Note: The secondary outcome was all-cause mortality, graft failure, rejection and eGFR ≥ 50%. Graft failure is defined as the development of kidney failure requiring

dialysis or KT, or death with a functional graft. Rejection is defined as biopsy-proven rejection.

[a] Multivariate analysis was adjusted for recipient and donor age, recipient and donor's sex, type of transplantation (living donor or deceased donor), history of diabetes mellitus, smoking history, and laboratory tests including hemoglobin, albumin, and estimated glomerular filtration rate at 1 year after kidney transplantation.

Abbreviations: HR; hazard ratio; CI, confidence interval; TSAT, transferrin saturation; eGFR, estimated glomerular filtration rate; KT, kidney transplantation.
